# Cholecystokinin Inhibits Inducible Nitric Oxide Synthase Expression by Lipopolysaccharide-Stimulated Peritoneal Macrophages

**DOI:** 10.1155/2014/896029

**Published:** 2014-07-13

**Authors:** Rafael Simone Saia, Fabíola Leslie Mestriner, Giuliana Bertozi, Fernando Queiróz Cunha, Evelin Capellari Cárnio

**Affiliations:** ^1^Departamento de Fisiologia, Faculdade de Medicina de Ribeirão Preto, Universidade de São Paulo, Avenida dos Bandeirantes 3900, 14049-900 Ribeirão Preto, SP, Brazil; ^2^Departamento de Farmacologia, Faculdade de Medicina de Ribeirão Preto, Universidade de São Paulo, 14049-900 Ribeirão Preto, SP, Brazil; ^3^Departamento de Enfermagem Geral e Especializada, Escola de Enfermagem de Ribeirão Preto, Universidade de São Paulo, 14040-902 Ribeirão Preto, SP, Brazil

## Abstract

Cholecystokinin (CCK) was first described as a gastrointestinal hormone. However, apart from its gastrointestinal effects, studies have described that CCK also plays immunoregulatory roles. Taking in account the involvement of inducible nitric oxide synthase- (iNOS-) derived NO in the sepsis context, the present study was undertaken to investigate the role of CCK on iNOS expression in LPS-activated peritoneal macrophages. Our results revealed that CCK reduces NO production and attenuates the iNOS mRNA expression and protein formation. Furthermore, CCK inhibited the nuclear factor- (NF-) *κ*B pathway reducing I*κ*B*α* degradation and minor p65-dependent translocation to the nucleus. Moreover, CCK restored the intracellular cAMP content activating the protein kinase A (PKA) pathway, which resulted in a negative modulatory role on iNOS expression. In peritoneal macrophages, the CCK-1R expression, but not CCK-2R, was predominant and upregulated by LPS. The pharmacological studies confirmed that CCK-1R subtype is the major receptor responsible for the biological effects of CCK. These data suggest an anti-inflammatory role for the peptide CCK in modulating iNOS-derived NO synthesis, possibly controlling the macrophage activation through NF-*κ*B, cAMP-PKA, and CCK-1R pathways. Based on these findings, CCK could be used as an adjuvant agent to modulate the inflammatory response and prevent systemic complications commonly found during sepsis.

## 1. Introduction

Innate immunity participates as first defense mechanism for host protection against microbial infections. Lipopolysaccharide (LPS) or endotoxin is a structural constituent of the Gram-negative bacterial wall, which is identified through a multiprotein complex composed by toll-like receptor- (TLR-) 4, CD4, and myeloid differentiation protein- (MD-) 2, triggering then the innate immune response [1, 2]. Despite its protective effects, the initial phase of experimental and clinical sepsis is characterized by “dysregulation of the inflammatory response” due to overproduction of cytokines and chemokines [3]. In this pathophysiological condition, the high levels of proinflammatory cytokines and the imbalance between the compensatory anti-inflammatory responses contribute to the disruption on microvascular integrity, tissue injury, evolution to shock, and development of multiple organ dysfunction syndrome [3, 4]. In part, these complications commonly may be explained by activation of leukocytes, such as monocytes/macrophages and neutrophils, which represent the major source for a large production of various inflammatory mediators [5, 6].

Among them, the exacerbated nitric oxide (NO) synthesis has been associated with diverse pathological sequelae of inflammation. NO is a gaseous and short-lived free radical, synthesized from the conversion of L-arginine in L-citrulline, and catalyzed by NO synthases (NOS) [7]. The two constitutive isoforms of NOS, neuronal (nNOS) and endothelial (eNOS), produce NO at such low levels, which acts as neurotransmitter and vasodilator [8], whereas the inducible isoform (iNOS) may also be expressed constitutively in some cell types; generally, it requires exposure to inflammatory stimuli, such as LPS, tumor necrosis factor (TNF)-*α*, and interferon (IFN)-*γ* [7, 9]. The iNOS is able to synthesize high quantities of NO (almost 1000-fold constitutive NOSs) and for long periods until its proteolytic degradation [10]. Moreover, the main regulatory mechanism for iNOS expression consists in the transcriptional level. The iNOS gene promoter region contains several consensus sequences for the binding of transcription factors, as NF (nuclear factor)-*κ*B, AP (activator protein)-1, CREB (cAMP-responsive element binding protein), and HIF (hypoxia-inducible factor) [11, 12]. The NF-*κ*B is a pleiotropic transcription factor and one of the most important for regulation of the iNOS synthesis, presented in the cytoplasm associated with its inhibitor, I*κ*B. The macrophage activation by LPS induces rapid phosphorylation and degradation of I*κ*B and dissociation of the complexes and allows subsequent nuclear translocation of NF-*κ*B [2, 12].

Cholecystokinin (CCK) was first described as a gastrointestinal hormone, released into the blood circulation by enteroendocrine I-cells when high levels of fatty acids or proteins reach this part of the intestine. CCK is synthesized in a pre-pro-polypeptide form and is posttranslationally modified, although the octapeptide, CCK-8, has been considered as a minor biologically active fragment. There are two receptors (CCK-Rs) identified, cloned, and responsible for CCK recognition, differing the affinity profile for gastrin or CCK and also tissue distribution [13]. The CCK-1R is mainly found in the periphery and shows higher selectivity for CCK, while CCK-2R is located in brain nuclei and gastric mucosa [13, 14]. However, apart from its role in digestion, CCK seems to be a promising therapeutic agent due to its anti-inflammatory properties demonstrated in different pathophysiological models [15–17]. Previously, we reported that increased plasma CCK levels during endotoxemic shock may be important to prevent cardiovascular collapse and tissue hypoperfusion [18]. Meanwhile the single-pulse pretreatment with low doses of exogenous CCK counteracts the hypotensive effects of LPS.

The identification of CCK-Rs expression in immune cells reinforced the hypothesis for an immunomodulatory role for this peptide [19, 20]. In pulmonary interstitial macrophages, both CCR-1R and -2R are expressed, they are functional biochemically and also their mRNA are upregulated by inflammatory stimuli with LPS [21]. Moreover, several functions in leukocytes may also be altered by CCK. In peritoneal macrophages [22] and neutrophils [23], the chemotaxis, phagocytic activity, and superoxide anions formation were reduced by incubation with different CCK concentrations. In relation to the synthesis of inflammatory mediators, the production of proinflammatory cytokines stimulated by LPS was attenuated when pulmonary interstitial macrophages were pretreated with CCK [24, 25], whilst the anti-inflammatory IL-10 was increased [18]. The involvement of this peptide on iNOS-derived NO synthesis, however, was not investigated in macrophages, despite the fact that we showed a significant reduction in iNOS expression in the aorta and liver in the endotoxemic rats [18].

This study was therefore undertaken to investigate whether CCK modulates the iNOS expression in LPS-activated peritoneal macrophages, as well as whether NF-*κ*B and/or cAMP-PKA signaling pathways were involved in affecting iNOS synthesis. Furthermore, we determined the expression of CCK-Rs in peritoneal macrophages and which receptor subtype may be responsible for these effects.

## 2. Materials and Methods

### 2.1. Animals

Experiments were performed on adult male Wistar rats weighing 180–220 g and of four to six weeks old. The animals were housed in a ventilated and controlled temperature chamber (25 ± 2°C) and exposed to a daily 12:12 h light-dark cycle (lights on at 0700 AM). They were housed in polypropylene cages of 4-5 rats per cage and given food and water* ad libitum* before beginning the experiment.

Animal handling followed the recommendations of the* Guide for the Care and Use of Laboratory Animals* [26, 27]. All procedures were conducted in accordance with the Ethical Guidelines of the School of Medicine of Ribeirão Preto, University of São Paulo, São Paulo, Brazil (protocol number 152/2009).

### 2.2. Drugs

Sulfated cholecystokinin octapeptide (CCK-8), lipopolysaccharide (LPS;* Escherichia coli *serotype O111:B4), aminoguanidine (AG), dexamethasone (DEX), pyrrolidine dithiocarbamate (PDTC), SQ-22,536, H-89, and forskolin were obtained from Sigma-Aldrich (St. Louis, MO, USA). The CCK-Rs antagonists, devazepide, and YM-022 were purchased from Tocris Bioscience (Ellisville, MO, USA).

### 2.3. Peritoneal Macrophage Culture

Rats were injected i.p. with 10 mL of thioglycolate medium (3% w/v; Sigma-Aldrich, St. Louis, MO, USA) to recruit macrophages into the peritoneal cavity. Four days after elicitation, the rats were euthanized by decapitation and the cells were collected through washing the peritoneum with cold RPMI 1640 medium. Macrophages were resuspended with RPMI 1640 medium supplemented with 10% heat-inactivated fetal bovine serum, 100 U/mL penicillin, and 100 *μ*g/mL streptomycin (Sigma) (complete medium), counted, and then cultured at densities 1–5 × 10^6^ cells/mL in 24-well plates. After incubation at 37°C on a humid heater with 5% of CO_2_ for 2 h, the nonadherent cells were removed washing each well once with PBS and twice with RPMI 1640 medium. The remaining cultured cells were at least 97% macrophages and trypan blue exclusion test showed viability higher than 95%.

### 2.4. Nitrite Determination

The stable product of NO oxidation, nitrite, was measured using the Griess reaction. Samples were mixed with an equal volume of the Griess reagent (2% sulfanilamide, 5% H_3_PO_4_, 0.2% naphthylethylenediamine dihydrochloride; Sigma) and incubated at room temperature for 10 min. The absorbance of each sample was measured spectrophotometrically at 540 nm and calculated comparing with various sodium nitrite concentrations (1.25, 2.5, 5, 10, 25, 50, 100, and 200 *μ*M) as a standard.

### 2.5. iNOS Real-Time PCR Analysis

Total mRNA was extracted from peritoneal macrophages adding 1 mL of TRIzol reagent (Sigma) per well using the acid guanidinium thiocyanate-phenol-chloroform extraction method, according to the manufacturer's instructions. mRNA was quantified using spectrophotometry at 260 nm (GeneQuant; Amersham Pharmacia Biotech, Fairfield, CT, USA) and the samples' purity was considered acceptable when the absorbance (260/280 nm) ratio was ≥1.8–2.0. Reverse transcription of total mRNA to cDNA was carried out with reverse transcription reaction (SuperScript II reverse transcriptase; Invitrogen Life Sciences, Grand Island, NY, USA). Target mRNA quantification was performed in an ABI 7500 Sequence Detection System using the SYBR-green Supermix fluorescence system (Applied Biosystems, Foster City, CA, USA) as indicated by the manufacturer. Rat primers pairs for iNOS and GAPDH (Sigma) were used for PCR amplification (cycling parameters: 3 min, 95°C; 15 s, 95°C; 60 s, 60°C, 40 cycle repetitions, and 1 min, 95°C) and were as follows: iNOS (forward, 5′-CTACCTACCTGGGGAACACCTGGG-3′ and reverse, 5′-GGAGGAGCTGATGGAGTAGTAGCGG-3′) (amplification product: 442 bases pair) and GAPDH (forward, 5′-GCCAAAAGGGTCATCATCTC-3′ and reverse, 5′-GGCCATCCACAGTCTTCT-3′) (amplification product: 226 pair bases). Single-product amplification was confirmed by melting curve analysis, submitting all samples to dissociation characteristics of double-stranded DNA during heating. Quantification was expressed in arbitrary units, and target mRNA levels were normalized to internal control GAPDH expression using the method of Pfaffl [28].

### 2.6. Western Blot

The macrophages were lysed in RIPA buffer (150 mM NaCl, 50 mM Tris, 1 mM EDTA, 1% Triton X-100, 0.1% sodium dodecyl sulphate, and 1% sodium deoxycholate) containing 4% (v/v) protease inhibitors cocktail (C*O*mplete, Roche Applied Science, Germany) and centrifuged (5000 g, for 10 min at 4°C) and then the supernatant was collected for analysis of iNOS, cytosolic NF-*κ*B p65, I*κ*B*α*, CCK-1R, and CCK-2R levels. The total protein concentration in lysates was measured using the colorimetric Bradford method (Bio-Rad Laboratories, Hercules, CA, USA).

Proteins (80 *μ*g) were separated by SDS-polyacrylamide gel electrophoresis (SDS-PAGE 10%) and transblotted onto nitrocellulose membranes (Amersham Pharmacia Biotech, Fairfield, CT, USA) (100 V for 1 h) using a transfer buffer (25 mM Tris, 192 mM glycine, and 20% methanol). After transfer was completed, blots were washed in Tris-buffered saline (TBS-T; 160 mM NaCl, 20 mM Tris, and 0.1% Tween 20) and incubated overnight at 4°C in blocking buffer (7% nonfat dry milk dissolved in TBS-T). Membranes were washed and incubated overnight at 4°C with one primary antibody, all diluted in TBS-T containing 5% nonfat milk: rabbit polyclonal anti-iNOS (1:5000; Sigma-Aldrich) (≈130 *κ*Da), rabbit polyclonal anti-NF-*κ*B p65 (1:500; Abcam, Cambridge, MA, USA) (≈60 *κ*Da), rabbit monoclonal anti-I*κ*B*α* (1:500; Cell Signaling, Danvers, MA, USA) (≈ 35 *κ*Da), rabbit polyclonal anti-CCK-1R (1:200; Santa Cruz Biotechnology, Dallas, TX, USA) (≈80 *κ*Da), rabbit polyclonal anti-CCK-2R (1:1000; Sigma and 1:50; Santa Cruz) (≈48 *κ*Da), mouse monoclonal anti-*β*-actin (1:3000; Abcam) (≈42 *κ*Da), or mouse monoclonal anti-*α*-tubulin (1:4000; Sigma) (≈55 *κ*Da). After these procedures, membranes were extensively washed to remove the unbound antibody excess and incubated for 1 h at room temperature with horseradish peroxidase-conjugated secondary antibodies, anti-rabbit (1:10000; Jackson ImmunoResearch, West Grove, PA, USA), or anti-mouse IgG (1:2000; Vector Laboratories, Burlingame, USA). The immunoreactive bands were visualized using enhanced chemiluminescence (ECL) reagents (Amersham) for 2 min and were digitally photographed (ChemiDoc XRS, Bio-Rad). The bands intensity was quantified using ImageJ software (National Institute of Health, Bethesda, MA, USA), which was normalized with the respective *β*-actin or *α*-tubulin signals.

### 2.7. ELISA for iNOS

The iNOS contents were determined using the method described previously by Marshall et al. [29] and modified by Saia et al. [18]. The macrophage homogenates were diluted in phosphate-buffered saline (PBS 0.1 M, pH 7.2) and incubated overnight at 4°C in Nunc Maxisorb plates (Life Technologies, Paisley, UK). The wells were washed with PBS containing 0.05% Tween-20 (PBS-T) and blocked with 1% (w/v) bovine serum albumin (BSA) for 1 h at room temperature. Subsequently, the polyclonal rabbit anti-iNOS antibody (1:1000; Sigma) was added for 1 h at room temperature. After washing, the biotinylated anti-rabbit IgG secondary antibody (1:200; Vector Laboratories) was added to each well, incubated with avidin-HRP (1:5000; Sigma) for 30 min, and the colour developed by addition of 3,3′,5,5′-tetramethylbenzidine substrate (TMB; BD Biosciences, Franklin Lakes, NJ, USA). The reaction was stopped with 2 N H_2_SO_4_ and the optical density (OD) reading at 450 nm was taken. The sample levels of iNOS were expressed as OD normalized to the respective total protein concentrations.

### 2.8. Nuclear Extract Preparation and Nuclear p65 Determination

The nuclear extracts were prepared from 10 × 10^6^cells using Nuclear Extraction kit (Cayman Chemical, Ann Arbor, MI, USA). Macrophages were gently scrapped, transferred to prechilled 15 mL conical tubes, and centrifuged (300 g, for 5 min at 4°C) and then the cell pellet resuspended in 5 mL of PBS containing phosphatase inhibitors cocktail (20 mM NaF, 1 mM *β*-glycerophosphate, and 1 mM Na_3_OV_4_). The cytosolic fraction was obtained by adding 500 *μ*L of cold hypotonic buffer [10 mM HEPES pH 7.5, 4 mM NaF, 10 *μ*M Na_2_MoO_4_, 0.1 mM EDTA, 20 mM NaF, 1 mM *β*-glycerophosphate, 1 mM Na_3_OV_4_, 0.1 mM 4-(2-aminoethyl)benzenesulfonyl fluoride (AEBSF), 5 *μ*M bestatin, 2 *μ*M leupeptin, 1.5 *μ*M N-(*trans*-epoxysuccinyl)-L-leucine 4-guanidinobutylamide (E-64), 1 *μ*M pepstatin A, and 0.8 nM aprotinin] to cell pellets and incubating for 15 min on ice. Thereafter, 100 *μ*L of NP-40 solution at 10% was added, mixed by pipetting, and microfuged (14000 g, for 30 s at 4°C) to the unlysed nuclei pellet. The supernatant (cytosolic fraction) was collected and stored at −80°C for analysis of p65 and I*κ*B*α* contents using western blot technique (as described above). The remaining nuclei were lysed in 50 *μ*L of cold nuclear extraction buffer (10 mM HEPES pH 7.9, 0.1 mM EDTA, 1.5 mM MgCl_2_, 420 mM NaCl, 10% glycerol, 0.1 mM AEBSF, 5 *μ*M bestatin, 2 *μ*M leucopeptin, 1.5 *μ*M E-64, 1 *μ*M pepstatin A, 0.8 nM aprotinin, 20 mM NaF, 1 mM *β*-glycerophosphate, 1 mM Na_3_OV_4_, and 1 mM DTT) and vigorously vortexed for 30 s and the tubes were gently rocked on ice for 15 min using a shaking platform. The same cycle was repeated once. The tubes were centrifuged (14000 g, for 10 min at 4°C) for cellular debris sedimentation, while the supernatant (nuclear fraction) was collected in prechilled tubes and frozen at −80°C.

The p65 quantification in nuclear extracts was obtained using ELISA kit according to the manufacturer's instructions (NF-*κ*B p65 Transcription Factor Assay, Cayman, USA). The sample levels were normalized to respective total protein concentrations determined by the Bradford assay and data was expressed as percentage relative to the control (saline) group.

### 2.9. cAMP Quantification

Total intracellular cAMP content was measured by commercial EIA kit according to the manufacturer's instructions (Cayman, USA). Peritoneal macrophages were cultured at density of 5 × 10^6^/well and lysed adding 200 *μ*L of HCl 0.1 N to each well and incubating at room temperature for 20 min. After scraping the mixture, the extracted samples were centrifuged (1000 g, for 10 min) for cell debris separation and the supernatant was stored at −80°C until analysis. Immediately before assaying, samples and standards (100 *μ*L) were acetylated adding 20 *μ*L of 4 M KOH and 5 *μ*L acetic anhydride in rapid succession, vortexed for 15 s, 5 *μ*L 4 M KOH more added and mixed in vortex again. The kit detection limit was 3 pmol/mL. The intracellular cAMP contents were normalized to respective total protein concentrations determined by the Bradford assay and data was expressed as pmol/mg total protein.

### 2.10. Statistical Analysis

All values are expressed as means ± S.E.M. Data shown in figures are representative of at least three independent experiments. All samples were measured in the same session to reduce experimental interassay variation. Data was compared using one-way analyses of variance (ANOVA) and significant differences were obtained using the Tukey multiple variances* post-hoc* test (GraphPad InStat version 3.0; GraphPad Software, San Diego, CA, USA). For all tests, statistical significance was considered when *P* < 0.05.

## 3. Results

### 3.1. CCK Inhibits LPS-Induced iNOS Expression in Peritoneal Macrophages

**Figure 1:**
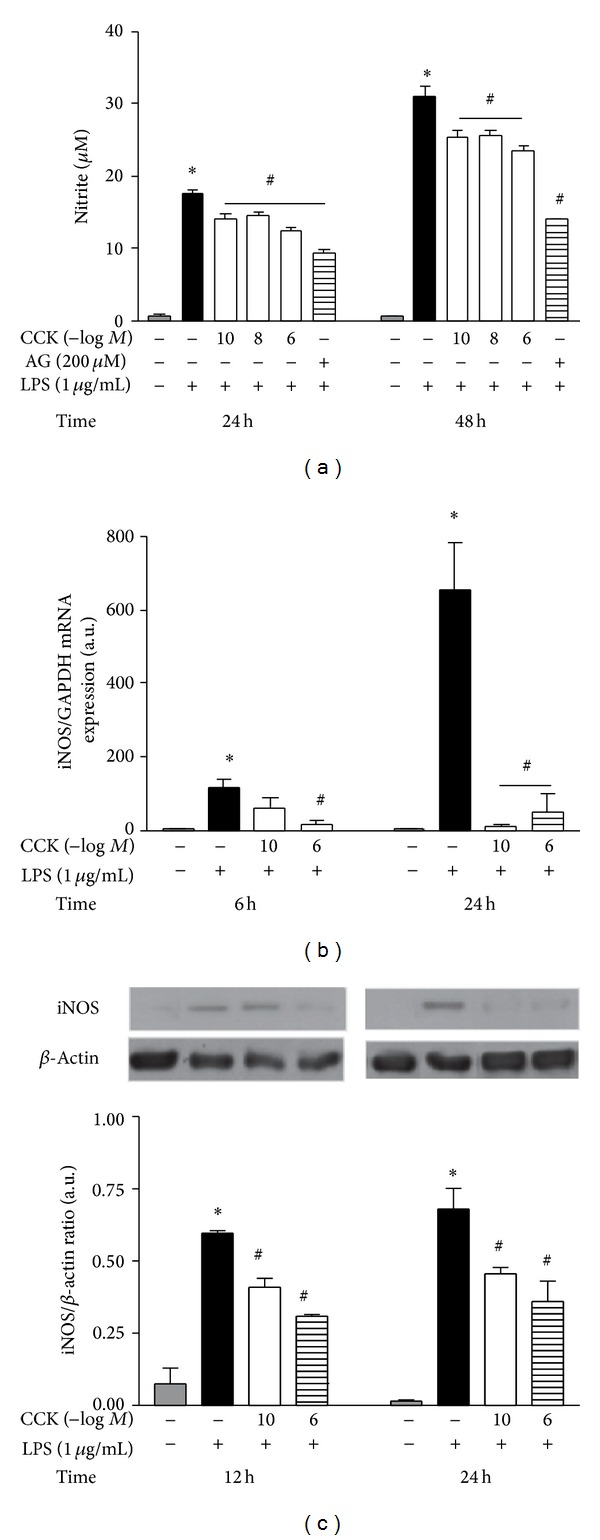
CCK modulates LPS-induced nitrite production and iNOS expression by peritoneal macrophages. The macrophage culture was pretreated with different CCK concentrations (10^−10^–10^−6 ^M) or aminoguanidine (200 *μ*M) for 30 min before the addition of LPS (1 *μ*g/mL) to the cell culture medium (time zero). At the indicated time-points, the culture supernatant was collected for nitritequantification by the Griess method (a), while adherent cells were harvested for iNOS expression determination. (b) Total mRNA was extracted and the expression of iNOS mRNA relative to GAPDH mRNA was analyzed by real-time PCR using specific gene primers. (c) Representative immunoblots and densitometry values (iNOS/*β*-actin ratio) of whole cell lysates. The *β*-actin levels were used as an internal control. Data is a representative of five independent experiments with similar results. Values are expressed as means ± S.E.M. *n* = 4–10 samples per group. ∗*P* < 0.05 versus saline and ^#^ *P* < 0.05 versus LPS group.

Figure 1 confirms the inducibility of iNOS gene requiring an initial inflammatory stimulus to start the synthesis of iNOS-derived NO. The macrophage culture incubation with LPS showed a slow and progressive increase in nitrite production, reaching the highest values at 48 h (30.88 ± 1.41 *μ*M) in comparison to vehicle-treated cells (0.65 ± 0.04 *μ*M; *P* < 0.05; Figure 1(a)). The formation of this product was associated with a significant induction on iNOS gene expression at 6 (*P* < 0.001), as well as 24 h after LPS stimulation (*P* < 0.001; Figure 1(b)). Furthermore, the protein synthesis was kept elevated at 12 (0.60 ± 0.01 versus 0.21 ± 0.08 arbitrary units; *P* < 0.001) and 24 h (0.68 ± 0.07 versus 0.02 ± 0.01 arbitrary units; *P* < 0.001) after LPS when compared to the control group (Figure 1(c)). The incubation with a selective iNOS inhibitor, aminoguanidine (AG), significantly reduced the supernatant nitrite concentration (Figure 1(a)).

On the other hand, the pretreatment of the macrophage culture with different concentrations of CCK inhibited the nitrite production. At 24 and 48 h post-LPS stimulation, a large range of CCK concentrations (10^−10^ to 10^−6^ M) exerted the anti-inflammatory ability reducing NO formation (Figure 1(a)). Subsequent* in vitro* studies were performed, however, choosing two CCK concentrations, 10^−10^ and 10^−6^ M. The lower was selected because it represents the most similar concentration found in plasma levels of endotoxemic rats (≈1000 pg/mL) [18], while the second could reproduce the initial high concentrations as a result of therapeutic CCK administration. After 24 h of incubation period, the CCK treatment at 10^−10^ M only slightly increased (31.25 ± 2.87 pg/mL; *P* > 0.05) the peptide concentration in the cell culture when compared to saline (22.4 ± 3.06 pg/mL) or LPS-treated macrophages (23.63 ± 2.38 pg/mL) (data not shown). Meanwhile, the higher tested CCK concentration maintained supraphysiological levels in the cell culture medium (>100 ng/mL; *P* < 0.001).

From the results demonstrating the modulatory role of CCK in LPS-induced nitrite production, we investigated the molecular mechanisms of action involved in this peptide. The peritoneal macrophages pretreated with both tested CCK concentrations were able to minimize the iNOS gene expression stimulated by LPS at 24 h, whereas only CCK at 10^−6^ M reduced it at 6 h (15.49 ± 10.80 arbitrary units; *P* < 0.05; Figure 1(b)). Furthermore, the presence of CCK at 10^−10^ M inhibited the iNOS synthesis in 31.67% at 12 h and 32.35% at 24 h in comparison to the LPS group (*P* < 0.05; Figure 1(c)). Accordingly, the CCK 10^−6^ M + LPS group showed decreased iNOS levels in 48.63% at 12 h (*F*_3,20_ =  43.73) and also 47.06% at 24 h (*F*_3,20_ = 49.96).

The CCK-treated macrophage culture did not change iNOS gene transcription or protein synthesis in the absence of an inflammatory stimulus such as LPS (data not shown).

### 3.2. CCK Inhibits NF-*κ*B p65 Translocation to Nucleus, Preventing Cytosolic I*κ*B*α* Degradation

**Figure 2:**
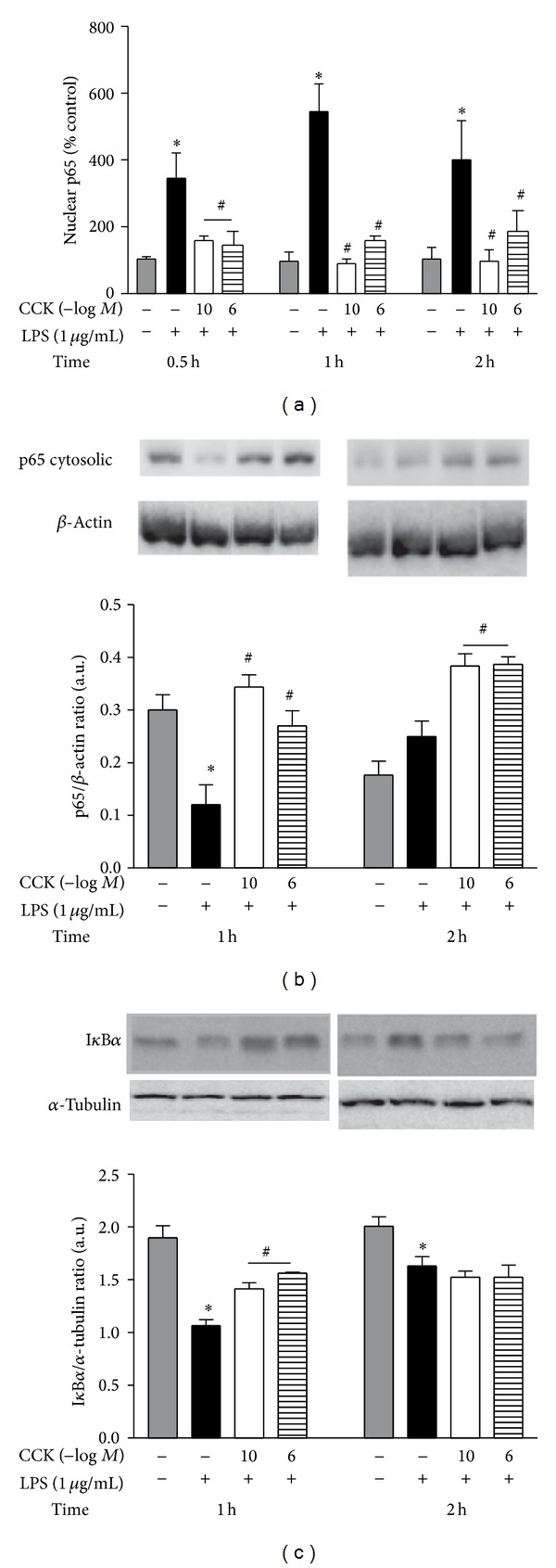
CCK reduces LPS-induced NF-*κ*B activation and p65 translocation to nucleus and also delays I*κ*B*α* degradation by peritoneal macrophages. The macrophage culture was pretreated with CCK (10^−10^ or 10^−6 ^M) for 30 min before the addition of LPS (1 *μ*g/mL) to the cell culture medium (time zero). At the indicated time-points, nuclear proteins were extracted for the quantification of p65 translocation by using ELISA kit (a). The nuclear p65 concentration was presented as the percentage relative to the control (saline) group. Cytosolic proteins were also extracted for the determination of p65 content and I*κ*B*α* degradation by western blot. Representative immunoblots with densitometry values were showed for p65/*β*-actin ratio (b) and I*κ*B*α*/*α*-tubulin ratio (c). The *β*-actin and *α*-tubulin levels were used as internal controls. Data is a representative of three independent experiments with similar results. Values are expressed as means ± S.E.M. *n* = 4–10 samples per group. ∗*P* < 0.05 versus saline and ^#^ *P* < 0.05 versus LPS group.

Peritoneal macrophages responded with a rapid p65 translocation to the nucleus induced by the inflammatory stimulus with LPS at 0.5, 1, and 2 h. Regarding the CCK effect on NF-*κ*B activation, it was observed that both peptide concentrations reduced the nuclear levels of p65 at all time-points investigated (Figure 2(a)). Interestingly, the CCK-pretreated cells returned their nuclear p65 content near to the control levels (at 1 h, LPS: 543.78 ± 84.57%, CCK 10^−10^ M + LPS: 90.42 ± 9.13%, CCK 10^−6^ M + LPS: 156.71 ± 16.76%; *F*_3,52_ = 32.11). In contrast at 1 h after LPS stimulation, the cytosolic p65 content in macrophages was significantly reduced when compared to control group (*P* < 0.05; Figure 2(b)). However, the LPS-induced decrease in the cytosolic p65 concentration was completely reverted by CCK addition to cell culture at 1 h (*F*_3,16_ = 17.22; *P* < 0.05) as also at 2 h (*P* < 0.05). In agreement, both tested CCK concentrations maintained high protein I*κ*B*α* levels at 1 h post-LPS stimulation in comparison to the exclusively LPS-treated cells, while there were no statistical differences amongst groups at 2 h (Figure 2(c)).

**Figure 3:**
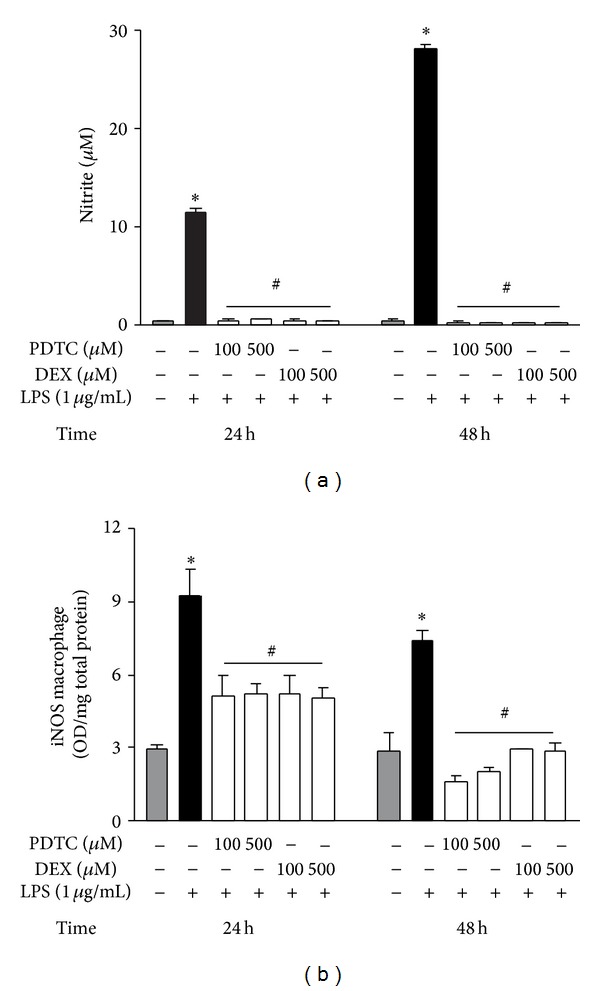
NF-*κ*B activation modulates LPS-induced iNOS expression by peritoneal macrophages. To demonstrate the importance of NF-*κ*B activation on LPS-induced iNOS expression, the macrophage culture was incubated with two different NF-*κ*B inhibitors [pyrrolidine dithiocarbamate (PDTC) and dexamethasone (DEX)] (100 and 500 *μ*M) for 30 min before the addition of LPS (1 *μ*g/mL) (time zero). The culture supernatant was collected for nitrite quantification by the Griess method (a), while the iNOS synthesis was determined in cell lysates by ELISA (b). Data is a representative of three independent experiments with similar results. Values are expressed as means ± S.E.M. *n* = 4–10 samples per group. ∗*P* < 0.05 versus saline and ^#^ *P* < 0.05 versus LPS group.

The importance of NF-*κ*B pathway in the LPS-induced iNOS expression was confirmed by preincubating the macrophage culture with two pharmacological agents, pyrrolidine dithiocarbamate (PDTC) and synthetic steroid dexamethasone (DEX). They reduced the nitrite production, as well as iNOS protein synthesis at all evaluated time-points (Figures 3(a) and 3(b)).

### 3.3. CCK Activates cAMP-PKA Pathway, Inhibiting iNOS Expression

**Figure 4:**
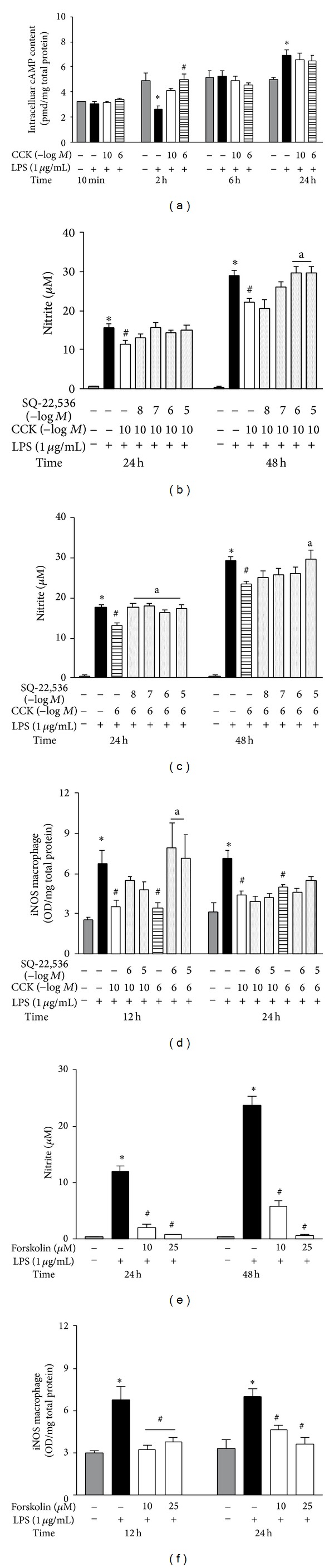
CCK increases cAMP formation which attenuates LPS-induced iNOS expression by peritoneal macrophages. The macrophage culture was pretreated with CCK (10^−10^ or 10^−6 ^M) for 30 min before the addition of LPS (1 *μ*g/mL) to the culture medium (time zero). At the indicated time-points, whole cell lysates were obtained for quantification of cAMP content by using ELISA kit (a), and samples were normalized with total protein concentration by Bradford method. To associate changes on intracellular cAMP with LPS-induced iNOS expression, the macrophage culture was incubated with an adenylyl cyclase inhibitor (SQ-22,536) (10^−8^ –10^−5 ^M) (b, c, and d) for 30 min before the addition of CCK at 10^−10^ or 10^−6^ M. Thirty minutes later, cells were stimulated by LPS. Additionally, the peritoneal macrophage culture was pretreated with an adenylyl cyclase activator (forskolin) (10 and 25 *μ*M) 30 min prior to LPS (e and f). The culture supernatant was collected for nitrite quantification by Griess method and the iNOS synthesis was determined in cell lysates by ELISA. Data is a representative of three independent experiments with similar results. Values are expressed as means ± S.E.M. *n* = 4–12 samples per group. ∗*P* < 0.05 versus saline, ^#^ *P* < 0.05 versus LPS, and ^a^ *P* < 0.05 versus respective CCK group.

Initially, we investigated whether peritoneal macrophages pretreatment with CCK changes the intracellular cAMP formation. The cell culture was stimulated with CCK (10^−10^ or 10^−6^ M) 30 min prior LPS and at various time intervals; macrophages were lysed for quantification of the cAMP content. LPS-induced inflammatory activation caused a reduction in the cAMP concentration at 2 h (2.57 ± 0.32 versus 4.92 ± 0.63 pmol/mg total protein; *F*_3,25_ = 7.34; *P* < 0.05), while there was an increase at 24 h (6.91 ± 0.40 versus 5.01 ± 0.18 pmol/mg total protein; *F*_3,20_ = 3.21; *P* < 0.05), both compared to their respective saline vehicle group (Figure 4(a)). Macrophage culture incubation with CCK at 10^−6^ M was solely responsible for the increase in the intracellular cAMP levels at 2 h after LPS (4.97 ± 0.46 pmol/mg total protein; *P* < 0.05). Moreover, the levels of this second messenger in CCK 10^−6^ M + LPS-treated macrophages were similar to the saline control group (*P* > 0.05). Although there is a tendency to increase in the cAMP concentration in the CCK 10^−10^ M + LPS group, this was not statistically different from LPS-treated cells. In the remaining time-points, no CCK concentration altered the intracellular cAMP content compared to the LPS group.

To prove the role of cAMP as the mechanism of action by CCK (especially at 10^−6^ M), we preincubated the cell culture with an adenylyl cyclase inhibitor, SQ-22,536. The pretreatment with this inhibitor did not modify the CCK effect at 10^−10^ M in reducing the nitrite production or iNOS synthesis at 24 h (Figures 4(b) and 4(d)). At 48 h, however, the presence of SQ-22,536 reverted the anti-inflammatory effect of CCK. Moreover, in the macrophage CCK 10^−6^ M + LPS group when adenylyl cyclase activity was blocked, a complete reversal in the nitrite production was observed at 24 h (Figure 4(c)). At 48 after LPS-stimulation, only the highest SQ-22,536 concentration was effective in blocking the CCK effect on NO synthesis. Accordingly, the adenylyl cyclase inhibition in CCK 10^−6^ M + LPS-treated cells showed a transient increase in the iNOS synthesis only at 12 h (*P* < 0.05; Figure 4(d)).

To determine whether an increase in the intracellular cAMP content could modify the NO synthesis in the peritoneal macrophages, the cell culture was exposed to different concentrations of forskolin, an adenylyl cyclase activator. The presence of forskolin caused a concentration-dependent reduction on nitrite production, as well as an iNOS expression induced by LPS at 12 and 24 h (Figures 4(e) and 4(f)).

**Figure 5:**
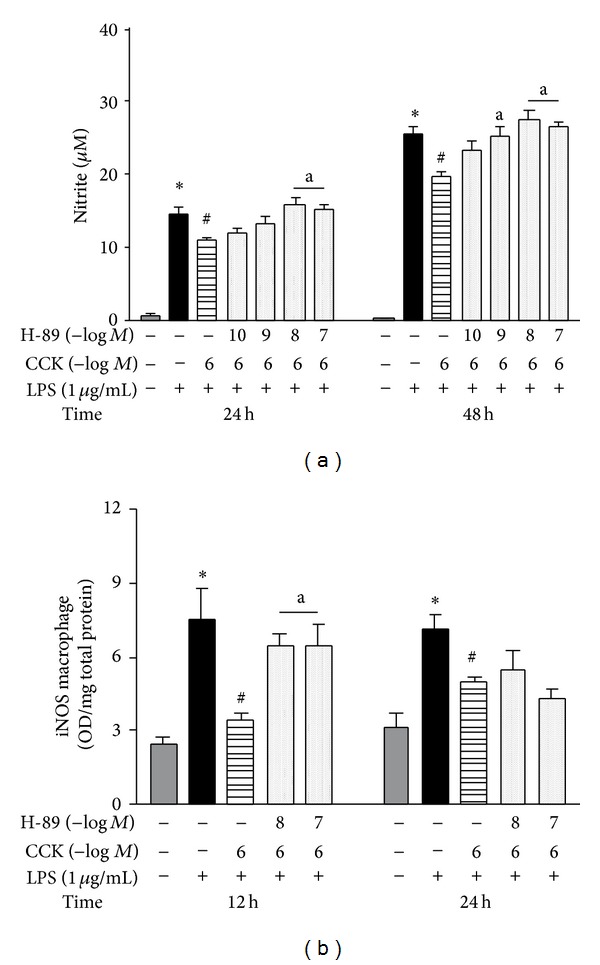
High CCK concentration activates PKA pathway which attenuates LPS-induced iNOS expression by peritoneal macrophages. To associate changes on intracellular cAMP and PKA activation with LPS-induced iNOS expression, the macrophage culture was incubated with PKA inhibitor (H-89) (10^−10^–10^−7 ^M) for 30 min before the addition of CCK at 10^−6 ^M (a and b). Thirty minutes later, cells were stimulated by LPS (1 *μ*g/mL) (time zero). The culture supernatant was collected for nitrite quantification by Griess method (a) and the iNOS synthesis was determined in cell lysates by ELISA (b). Data is a representative of three independent experiments with similar results. Values are expressed as means ± S.E.M. *n* = 4–12 samples per group. ∗*P* < 0.05 versus saline, ^#^ *P* < 0.05 versus LPS, and ^a^ *P* < 0.05 versus respective CCK group.

To evaluate whether CCK acts through the activation of PKA pathway, we performed selective inhibition with H-89. The preincubation with H-89 in the macrophage culture blocked the CCK effect to reduce the nitrite production, at 24 and 48 h (Figure 5(a)). Furthermore, the H-89 addition in the culture increased the iNOS synthesis at 12 h after LPS (6.4 ± 0.48 OD/mg total protein) compared to the CCK 10^−6^ M + LPS group (3.4 ± 0.38 OD/mg total protein; *F*_4,65_ = 19.74; *P* < 0.05; Figure 5(b)). As with SQ-22,536, the H-89 pretreatment alone did not change the LPS-stimulated NO production in the absence of CCK (data not shown).

### 3.4. CCK-1R Is Upregulated by LPS and CCK and Is Responsible for CCK Inhibition in iNOS Expression

**Figure 6:**
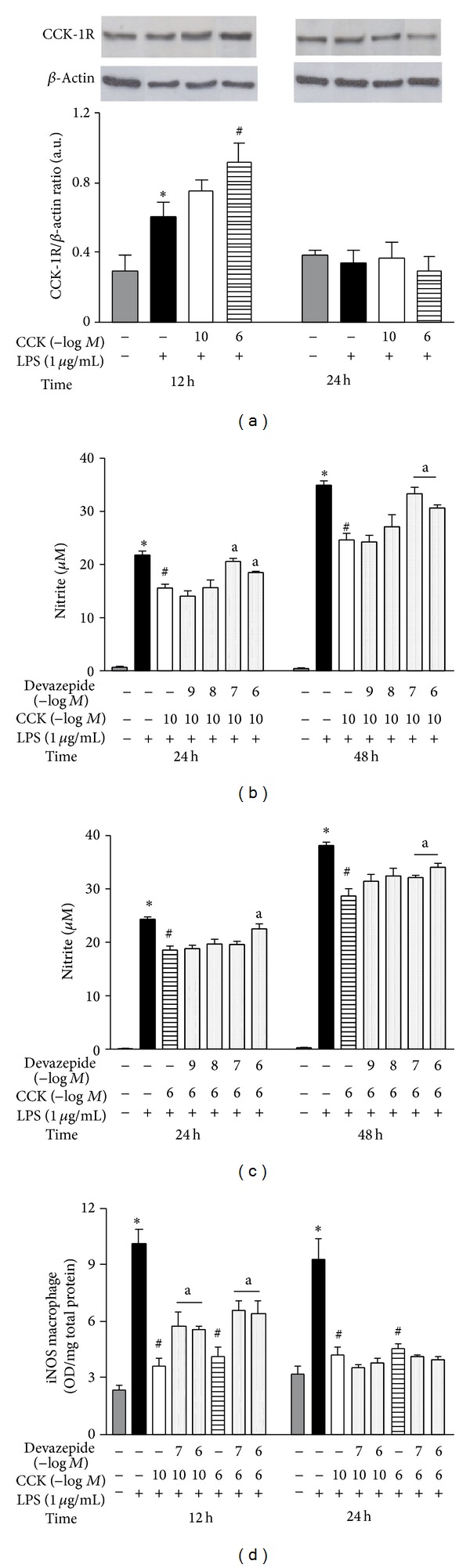
CCK attenuates LPS-induced iNOS expression interacting predominantly through CCK-1R by peritoneal macrophages. The macrophage culture was pretreated with CCK (10^−10^ or 10^−6 ^M) for 30 min before the addition of LPS (1 *μ*g/mL) to the culture medium (time zero). At the indicated time-points, whole cell lysates were obtained for analysis of CCK-Rs expression by western blot. (a) Representative immunoblots with densitometry values were shown for CCK-1R/*β*-actin ratio. The *β*-actin levels were used as an internal control. The relevance of CCK-1R subtype was pharmacologically tested, incubating macrophage culture with selective antagonist [devazepide (CCK-1R antagonist; 10^−9^–10^−6^ M)] for 30 min before the addition of CCK at 10^−10^ or 10^−6^ M. Thirty minutes later, cells were stimulated by LPS. The culture supernatant was collected for nitrite quantification by Griess method (b and c) and the iNOS synthesis was determined in cell lysates by ELISA (d). Data is a representative of three independent experiments with similar results. Values are expressed as means ± S.E.M. *n* = 4–12 samples per group. ∗*P* < 0.05 versus saline, ^#^ *P* < 0.05 versus LPS, and ^a^ *P* < 0.05 versus respective CCK group.

The CCK-Rs have been identified in immune cells [19–21]; however, in peritoneal macrophages there is no data demonstrating whose subtype is predominant or whether the expression of these receptors changes after incubating with endotoxin or CCK. These macrophages showed CCK-1R-like immunoreactivity localized near the plasmatic membrane in the control group, which may be intensified after LPS stimulation (data not shown). On the other hand, no suggestive detection of CCK-2R was observed, regardless of the treatment or the time after incubation. Subsequently by using western blot technique, CCK-1R showed an intense expression level (Figure 6(a)), while CCK-2R was not detected in whole macrophage lysates (despite testing different manufacturer's antibodies or protein quantity). Figure 6(a) showed that LPS-stimulation increased transiently the synthesis of CCK-1R in comparison to control group at 12 h (0.61 ± 0.08 versus 0.30 ± 0.09 arbitrary units; *P* < 0.05), but not at 24 h (0.34 ± 0.07 versus 0.39 ± 0.03 arbitrary units; *P* > 0.05). Moreover, it may also be noted that CCK-1R expression was elevated when macrophages were treated with both CCK concentrations, but only the CCK 10^−6 ^M + LPS group showed a statistical difference compared to the exclusive LPS-treated cells (0.91 ± 0.12 arbitrary units; *F*_3,20_ = 16.75; *P* < 0.05; Figure 6(a)). In contrast, at 24 h no difference between treatments was found and the expression of CCK-1R induced by LPS returned close to control levels.

Since peritoneal macrophages express CCK-1R, we pharmacologically evaluated whether CCK inhibits the iNOS-derived NO production mediated by this receptor. The culture was preincubated with a selective CCK-1R antagonist, devazepide, 30 min before CCK treatment. Different antagonist concentrations were able to reverse the inhibitory effect of CCK on nitrite production at 24 and 48 h after LPS-stimulation (Figures 6(b) and 6(c)). In addition, the increased nitrite formation found during macrophage incubation with devazepide may be related to the concomitant increased iNOS synthesis at 12 h (Figure 6(d)).

**Figure 7:**
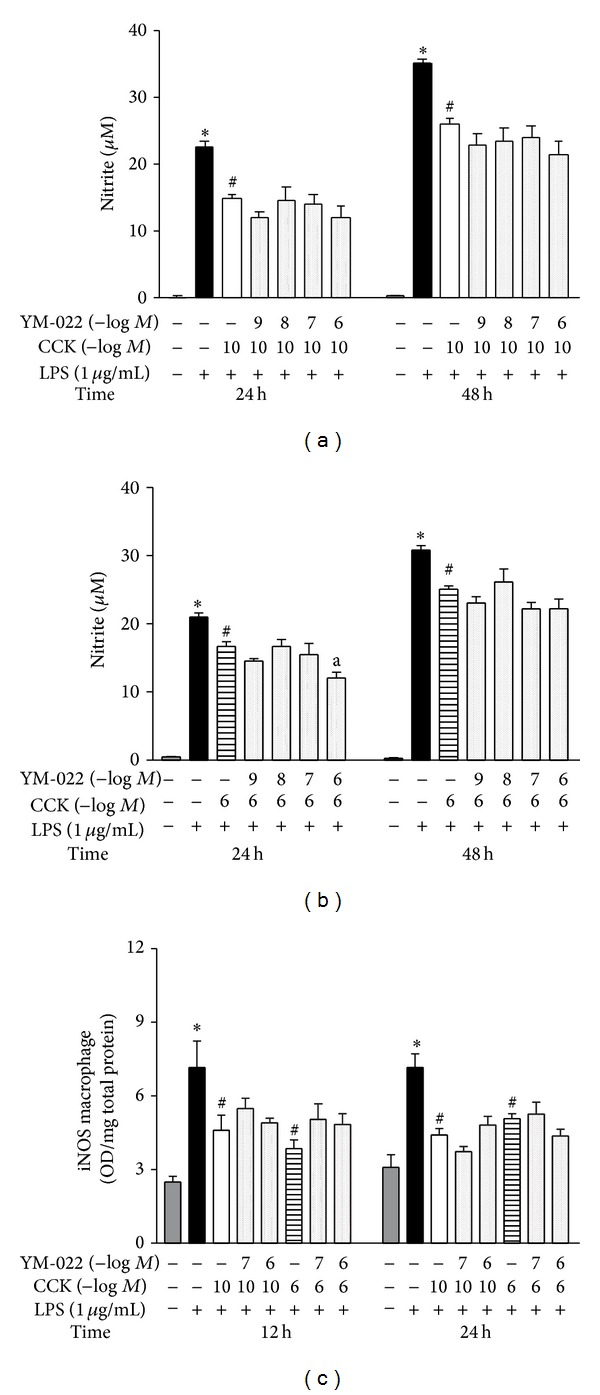
CCK-2R does not alter LPS-induced iNOS expression by peritoneal macrophages. The relevance of CCK-2R was pharmacologically tested, incubating macrophage culture with selective antagonist [YM-022 (CCK-2R antagonist; 10^−9^–10^−6 ^M)] for 30 min before the addition of CCK at 10^−10^ or 10^−6 ^M. Thirty minutes later, cells were stimulated by LPS (1 *μ*g/mL) (time zero). At the indicated time-points, the culture supernatant was collected for nitrite quantification by Griess method (a and b), while the iNOS synthesis was determined in cell lysates by ELISA (c). Data is a representative of three independent experiments with similar results. Values are expressed as means ± S.E.M. *n* = 4–12 samples per group. ∗*P* < 0.05 versus saline, ^#^ *P* < 0.05 versus LPS, and ^a^ *P* < 0.05 versus respective CCK group.

Despite the fact that the CCK-2R expression was not detected by the techniques employed in our study, we tested the pharmacological participation of this receptor in the CCK effect on NO production. As was done with devazepide, the culture was preincubated with different concentrations of a highly selective CCK-2R antagonist, YM-022. Figures 7(a) and 7(b) showed that nitrite concentration in the supernatant was not changed independently of the time-point evaluated or the antagonist concentration tested. Accordingly, YM-022 at 10^−7^ and 10^−6^ M did not affect the iNOS synthesis compared to CCK-treated cells (Figure 7(c)).

Furthermore, devazepide or YM-022 pretreatment did not modify the supernatant nitrite concentration with or without inflammatory stimulus by LPS (data not shown).

## 4. Discussion

In this study, we demonstrated that the gastrointestinal hormone CCK inhibits LPS-stimulated iNOS mRNA expression and protein synthesis in peritoneal macrophages. The immunomodulatory mechanism for this peptide may be associated with inhibition of the NF-*κ*B signaling pathway, caused by the reduction in I*κ*B*α* degradation and decreased p65 translocation to the nucleus. Moreover, CCK also increases intracellular cAMP content and activates PKA pathway, reducing the iNOS expression and nitrite production. Additionally, CCK-1R is highly expressed in peritoneal macrophages and may be upregulated by LPS and high CCK concentrations. The pharmacological blockage studies confirmed CCK-1R as the main receptor responsible for CCK recognition and attenuation in iNOS synthesis.

During experimental and clinical sepsis, the circulating endotoxin and other microbial components are responsible for activation of phagocytic cells, triggering them to express iNOS mRNA and synthesize large NO amounts [5, 30, 31]. Since macrophages and monocytes express CCK-Rs [19, 20], a possible regulatory role for CCK on cellular and humoral innate immunity has been questioned. Previous studies reported that CCK administration and endogenous stimulation for CCK secretion by applying a model of lipid-enriched enteral nutrition reduced TNF-*α*, IL-1*β*, and IL-6 concentrations in plasma, lung and cardiac, hepatic, and splenic tissues [32, 33]. In agreement with the previous part, CCK inhibits membrane CD14 expression and also the IL-1*β* synthesis and mRNA expression by LPS-activated pulmonary interstitial macrophages [24, 34]. Excepting the synthesis of proinflammatory cytokines, an increased IL-10 production was found when endotoxemic rats and also LPS-stimulated peritoneal macrophages were pretreated with low CCK concentrations [18]. Furthermore, we showed that LPS-induced nitrite formation was reduced by incubating peritoneal macrophage culture with different CCK concentrations and it could be mediated by a self-regulatory IL-10 mechanism [18]. However, the proposed molecular mechanisms regarding how CCK inhibits iNOS-derived NO synthesis remain unclear. In the present study, we elucidated that attenuation of nitrite production by CCK occurs at the transcriptional level, suppressing iNOS mRNA expression and also leading to diminished protein synthesis. Our* in vitro *results confirmed the animal findings that CCK administration before the endotoxemia induction in a single pulse [15, 32, 35] and near at the physiological levels exerts anti-inflammatory properties, reducing the iNOS expression and cytokines synthesis [18].

In septic patients and experimental endotoxemia, the nuclear NF-*κ*B binding activity in peripheral blood mononuclear cells represents a valuable marker, predicting fatal outcome, tissue injury, and also amplification of inflammatory response [36, 37]. The activation of the NF-*κ*B signaling pathway is fundamental for the transcription of several proinflammatory genes, including iNOS [12]. The deletion of consensus sequences for the NF-*κ*B binding in the iNOS promoter region severely compromises the maximal inducibility of its gene in LPS-treated macrophages [11]. Therefore, we investigated whether CCK could mediate the downregulation on iNOS expression by altering the NF-*κ*B pathway activation. Our data positively confirmed this hypothesis, whereas peritoneal macrophages pretreated with CCK showed the reduction on nuclear p65-dependent DNA-binding activity induced by LPS. Additionally, those CCK-treated cells presented minor I*κ*B*α* degradation, which is an inhibitory protein that interacts with NF-*κ*B dimers and sequestrates this transcription factor in inactive cytosolic form [2]. In LPS-stimulated pulmonary interstitial macrophages, the CCK treatment declined the NF-*κ*B binding activity in the nuclear fraction in a concentration-dependent manner [24]. In accordance with what is presented in our study, this other resident macrophage subpopulation exhibited increased I*κ*B*α* and also reduced phosphorylated I*κ*B*α* levels, suggesting an inhibitory role for CCK on the proteolytic degradation of this protein [24]. Similar findings have been identified* in vivo* during endotoxemic shock, where lung and spleen I*κ*B*α* expression levels were maintained elevated by treating rats with high CCK doses [35]. Moreover, CCK prevented NF-*κ*B p65 translocation to the nucleus, expression of proinflammatory cytokines, adhesion molecule (ICAM-1), and macrophage influx (CD68 mRNA expression) in the kidney cortex of diabetic rats [16].

The second messenger cAMP has been recognized as an innate immunity regulator, affecting phagocytosis, inflammatory response, and the synthesis of reactive oxygen species. However, intracellular cAMP formation may be modified depending on the inflammatory stimuli, as well as the cellular type. In peripheral blood mononuclear cells from severe sepsis patients [38], basal and stimulated cAMP accumulation is compromised, while LPS attenuated the mRNA expression of several adenylyl cyclase isoforms in different rat tissues, alveolar macrophages, and Küpffer cells [39]. On the other hand, adenylyl cyclase activity was induced by LPS in murine macrophages through a NF-*κ*B-dependent pathway [40]. In the present study, the intracellular cAMP content demonstrated a biphasic profile in LPS-stimulated peritoneal macrophages. The rapid downregulation on the expression of adenylyl cyclase isoforms may be responsible for reducing the cAMP concentration at 2 h. Moreover, the higher intracellular cAMP content observed at 24 h may be the consequence of macrophage sensitization [40] or the decrease in phosphodiesterase activity induced not only by LPS itself [41], but also by IL-10 dependent mechanism [42]. Regarding the iNOS expression, we confirmed that adenylyl cyclase activation by forskolin abolished LPS-induced nitrite production and iNOS expression. Our data is in accordance with previous reports, demonstrating that forskolin and also cAMP analogs reduced iNOS mRNA expression as well as protein synthesis, possibly preventing the I*κ*B degradation and NF-*κ*B translocation to the nucleus [43, 44].

Several immune cells, including neutrophils [23], lymphocytes, and macrophages [22], showed increased intracellular cAMP formation stimulated by CCK. Furthermore, this increment is rapid but transient lasting only few minutes. In association with the inflammatory LPS challenge, CCK potentiates cAMP production and also PKA activity in pulmonary interstitial macrophages [24]. These effects are related to decreased IL-1*β* mRNA expression and inhibition on the NF-*κ*B pathway through minor I*κ*B*α* degradation [24]. In our study using peritoneal macrophages, the intracellular cAMP content did not change at 10 minutes and 6 h, independently of CCK or LPS treatments. However, only the higher CCK concentration at 10^−6^ M was responsible for restoring the cAMP levels at 2 h and at values close to the control group. Moreover, this increase contributed to inhibiting iNOS expression, nitrite production, and activating PKA-dependent pathway in those macrophages. Since the increment of cAMP content was temporally later than the inhibition of NF-*κ*B translocation, in our study we speculate that CCK exerts its anti-inflammatory role through these two mechanisms independently. The regulatory mechanisms between cAMP-PKA pathway and iNOS-derived NO synthesis are explained by the inhibition of transcription factors activation (including NF-*κ*B), iNOS mRNA expression and posttranslational protein stability [43, 44], suppression on proinflammatory cytokines, and enhancement on anti-inflammatory mediators production (such as IL-10) [45].

The alterations on functions and activation of macrophages by CCK are mediated through interaction with two main G protein-coupled receptors, CCK-1R and -2R [13, 14, 17, 19]. Furthermore, the CCK-Rs in these cells demonstrated some particularities, including rapid internalization induced by agonists, high affinity, and lower density compared to pancreas acinar cells [21, 46]. Interestingly, both receptors may be upregulated for up to 12 h after LPS stimulation, being CCK-2R mRNA expression quantitatively higher than CCK-1R [21]. This evidence reinforces the biological immunomodulatory role for this peptide during inflammatory conditions, suggesting that macrophages become more responsive to CCK. Nevertheless, no previous studies identified expression of the CCK-Rs in rat peritoneal macrophages and what subtype contributes to anti-inflammatory activity of this peptide. Here, we found that CCK-1R expression occurred at higher levels and is upregulated at 12 h after incubation with LPS. Surprisingly, the CCK treatment at 10^−6^ M prior LPS also increased transiently the CCK-1R expression, contradicting the downregulation in pancreatic tissue after chronic [47] and intermittent CCK infusion [48]. In relation to CCK-2R expression, no detectable levels were found using the western blot technique regardless of treatment and time of incubation, suggesting that it is expressed at very low levels in this macrophage subpopulation. The CCK-2R however has already been identified in pulmonary macrophages, lymphocytes, and leukemic cells [20, 21]. Based on these results, we pharmacologically investigated the contribution of each receptor in the inhibitory CCK role in iNOS-derived NO synthesis. As expected, only the selective CCK-1R antagonist, devazepide, increased nitrite production and also iNOS expression, therefore reverting the CCK effect. The pretreatment with YM-022, a highly selective CCK-2R, did not modify nitrite concentration neither in the culture supernatant nor in the iNOS levels. Our results are in agreement with CCK-Rs expression and demonstrated that the CCK-1R subtype could be the major receptor involved in the anti-inflammatory effect of CCK. In pulmonary macrophages, similar findings showed that the CCK-1R antagonist was more efficient in blocking the CCK effect, modulating NF-*κ*B, cAMP-PKA, and p38 kinase pathways and leading to increased IL-1*β* mRNA expression [24].

## 5. Conclusions

In summary, our data demonstrate that LPS-stimulated peritoneal macrophages are responsive to the anti-inflammatory properties of the gastrointestinal hormone CCK. This peptide reduces iNOS-derived NO synthesis at the translational level and also modulates different intracellular signaling pathways. The NF-*κ*B activation is inhibited, through reduced I*κ*B*α* degradation and subsequently minor p65-dependent translocation to the nucleus. Moreover, the activation of the cAMP-PKA pathway by CCK results in a modulatory role in iNOS expression and nitrite production. In peritoneal macrophages, differing from other resident cells, the major receptor subtype is the CCK-1R, whose expression levels are increased by LPS stimulation. The most important finding of the present study is that very low CCK concentration exerts anti-inflammatory properties. These data suggest that CCK might be used as a promising adjuvant therapeutic agent, preventing the “dysregulation of the inflammatory response” and the systemic complications usually described during sepsis.
